# Complete Genome Sequence of *Starkeya* sp. Strain ORNL1, a Soil Alphaproteobacterium Isolated from the Rhizosphere of Populus deltoides

**DOI:** 10.1128/MRA.00644-20

**Published:** 2020-07-02

**Authors:** Mircea Podar, Joel Turner, Leah H. Burdick, Dale A. Pelletier

**Affiliations:** a Biosciences Division, Oak Ridge National Laboratory, Oak Ridge, Tennessee, USA; University of Southern California

## Abstract

*Starkeya* sp. strain ORNL1 is an alphaproteobacterium isolated from the rhizosphere of an Eastern cottonwood tree. *Starkeya* spp. are physiologically versatile, using a wide range of nutritional and energetic resources and serving important ecological roles in carbon and sulfur cycling. The 6.3-Mb chromosome of *Starkeya* sp. strain ORNL1 was completely sequenced and will help in understanding nutrient cycles.

## ANNOUNCEMENT

*Starkeya* is a genus of alphaproteobacteria classified into the family *Xanthobacteraceae*, most closely related to *Ancylobacter* (1, 2). Its type species, Starkeya novella, was isolated from soil in the mid-1930s and was originally classified as *Thiobacillus* (1, 3). A second formally described species, S. koreensis, was isolated from rice straw (4). *S. novella* has been studied extensively, being able to grow as a facultative chemoautotroph, both consuming and producing CO_2_ (5–7), utilize a variety of C_1_ substrates (methanol, formate, formamide) (8, 9), and oxidize sulfur compounds (9, 10).

**FIG 1:**
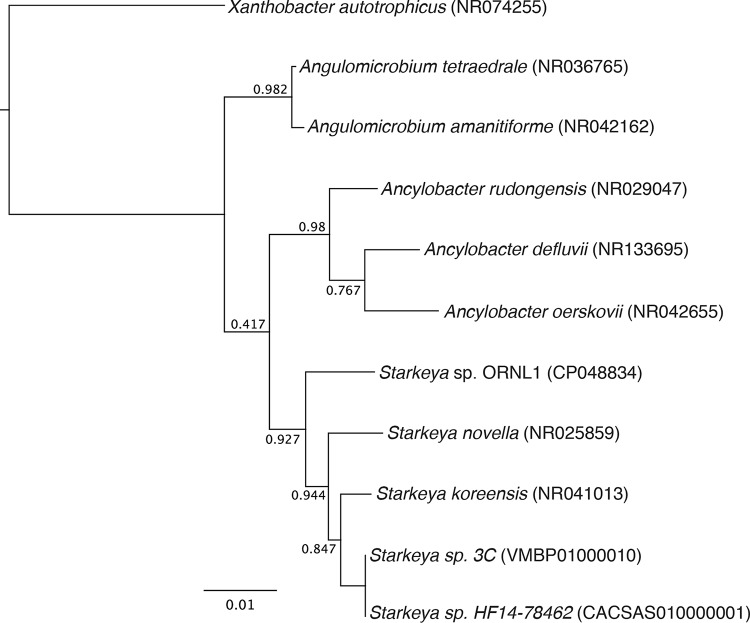
Phylogenetic tree (FastTree) of *Starkeya* sp. strain ORNL1 and related bacteria based on SSU rRNA genes. Reference sequences were obtained from GenBank; accession numbers are in parentheses. The alignment was edited by removing heterogeneous-length ends. The tree was rooted with *Xanthobacter*. Numbers at the nodes indicate support values.

Here, we report the complete genome sequence of a putative novel species of *Starkeya*, strain ORNL1, isolated from the microbial rhizosphere of an Eastern cottonwood tree (Populus deltoides) in Oak Ridge, Tennessee. We used flow cytometry to deposit single bacterial cells from a microbially enriched rhizosphere fraction onto Reasoner’s 2A (R2A) agar (11, 12). Colonies that developed upon incubation at 28°C were identified taxonomically by small-subunit (SSU) rRNA gene amplification and direct Sanger sequencing using the universal primers 27F and 1492R (13). Default parameters were used for all software used for sequence data analysis unless otherwise specified. Sequences were assembled and manually trimmed based on quality in Geneious R11 (14), and close relatives were identified by MegaBLAST (15) against the NCBI rRNA database. The sequences and their top relatives were aligned with ClustalW 2.1 (16) in Geneious, and the alignment was manually edited to remove heterogeneous ends. One colony was identified as representing a *Starkeya* sp., sharing 98% pairwise sequence identity with the rRNA sequences of *S. novella* and *S. koreensis*. Phylogenetic analysis using FastTree 2.1.11 (17) indicated that the novel isolate, designated *Starkeya* sp. strain ORNL1, is most closely related to *S. novella* (Fig. 1).

For PacBio genomic sequencing, *Starkeya* sp. strain ORNL1 was grown in liquid R2A medium at 30°C for 2 days. Genomic DNA was purified using the Quick-DNA fungal/bacterial miniprep kit (Zymo Research) and fragmented to an average size of 10 kb using g-TUBEs (Covaris, Woburn, MA). A sequencing library was constructed using a SMRTbell template prep kit 1.0 (Pacific Biosciences, Menlo Park, CA) and sequenced on a Pacific Biosciences Sequel instrument. The sequences were filtered based on quality and assembled using the software HGAP4 in PacBio SMRTLink 7.0, using a target genome size of 6 Mbp, a minimum subread length of 500, a minimum concordance of 85%, and a seed coverage of 30-fold, with the “aggressive” option on. In all, 64,603 filtered subreads (*N*_50_, 10,183 nucleotides [nt]) were assembled into three polished contigs totaling 6,326,767 nt, with a mean coverage of 350-fold and a G+C content of 66%. We predicted the genes using Prokka (18) and performed *de novo* assembly in Geneious 11 (14), together with PacBio spanning sequence reads. All three contigs were self-assembled, and based on gene calls, we identified overlapping assembly ends that enabled closing of the genome as a single, circular chromosome of 6,286,188 bp. Gene prediction and functional annotation were performed using the NCBI Prokaryotic Genome Annotation Pipeline (PGAP) 4.8 (19), which identified 5,826 protein coding sequences, 48 tRNAs, 2 rRNA operons, and 13 other noncoding or regulatory RNAs (ncRNAs). We also generated a metabolic model in KBase (20), accessible at https://narrative.kbase.us/narrative/55377, together with its RAST annotation. The average nucleotide identity (ANI) of 83% relative to *S. novella*, calculated with FastANI 0.1.2 (21), suggests that *Starkeya* sp. strain ORNL1 may represent a novel species, provisionally referred to as Starkeya rhizosphaerae ORNL1. Its genome is significantly larger than that of *S. novella* (4.7 Mbp) and will aid in understanding adaptation to the rhizosphere microbiome community, as well as uncovering potential broader metabolic versatility in this group of bacteria.

### Data availability.

The *Starkeya* sp. strain ORNL1 genome sequence has been deposited in GenBank under the accession number CP048834. The version described in this paper is the first version, CP048834.1. The PacBio reads have been deposited in the SRA under the accession number SRX7858655.
